# Supplementary material for: Dual receptor-sites reveal the structural basis for hyperactivation of sodium channels by poison-dart toxin batrachotoxin
Source: Nat Commun. 2024 Mar 14;15:2306. doi: 10.1038/s41467-024-45958-w (PMC10940626; doi:10.1038/s41467-024-45958-w)
Supplement: Supplementary file 1 — Supplementary Information [file 41467_2024_45958_MOESM1_ESM.pdf]

Supplementary Information for:

Dual receptor-sites reveal the structural basis for hyperactivation of sodium channels by poison-dart toxin batrachotoxin

Lige Tonggu[1,7], Goragot Wisedchaisri[1,7], Tamer M. Gamal El-Din[1,7], Michael J. Lenaeus[2], Matthew M. Logan[3,5], Tatsuya Toma[3,6], Justin Du Bois[3], Ning Zheng[1,4,*], and William A. Catterall[1,*]

[1]Department of Pharmacology, University of Washington, Seattle, WA, 98195, USA
[2]Division of General Internal Medicine, Department of Medicine, University of Washington, Seattle, WA, 98195, USA
[3]Department of Chemistry, Stanford University, Stanford, CA, 94305, USA
[4]Howard Hughes Medical Institute, University of Washington, Seattle, WA, 98195, USA
[5]Present address: Vividion Therapeutics, Inc., 5820 Nancy Ridge Dr., San Diego, CA, 92121, USA
[6]Present address: PRISM BioLab Co., Ltd., 2-26-1 Muraokahigashi, Fujisawa-shi, Kanagawa, 251-8555, Japan
[7]These authors contributed equally: Lige Tonggu, Goragot Wisedchaisri, Tamer M. Gamal El-Din
*Corresponding authors

The file includes Supplementary Discussion, Supplementary Figs. 1-11, and Supplementary Tables 1-4.

Supplementary Discussion

The N-terminal domain

The N-terminal domain (NTD) and the intracellular region of the *D*I-S6 segment are essential for the function of Na_V1.5 as mutations in this region cause cardiac arrhythmia in humans. Our cryo-EM density map of the NTD and the cytoplasmic *D*I-S6 region is resolved to ~3.5-4.0 Å (Fig. 1e). Although the density for the side chains in these regions are often lacking (Supplementary Fig. 3a, b), our structure modeling and refinement nevertheless suggested several van der Waals contacts made by residues between the NTD and the cytoplasmic *D*I-S6 (Supplementary Fig. 3a). In addition, a hydrogen bond between Ser62 of the NTD and Ser235 of *D*I S4-S5 linker (Supplementary Fig. 3c) suggested a role of the NTD in the voltage-dependent activation of the channel via a conformational stabilization of the S4-S5 linker in the activated conformation induced by the binding of BTX-B. Mutations in the NTD of the human Na_V1.5 gene are associated with cardiac arrhythmia phenotypes including long QT syndrome 3 (LQT3; G9V, R18Q*, R27H, E30G*, R43Q, E48K*, P52S*, R53Q*, R104G*, S115G*) and Brugada syndrome (BRGDA1; R18Q*, R27H, N70K*, D84N*, F93S*, I94S*, V95I, R104Q*, R104W*, N109K*). (*Unknown pathological significance) The locations of these mutations can be mapped on to our structural model of the NTD (Supplementary Fig. 3d).

The junction between the NTD and the cytoplasmic *D*I-S6 is also an important location for post-translational modification of Na_V1.5. Residue K442 (human Na_V1.5 numbering) has been shown to be a target for SUMOylation by SUMO1 that increased the inward sodium current (Yoon et al. BioRxiv 2022). The equivalent rNa_V1.5 K443 is located at the end of the cytoplasmic *D*I-S6 helix that is partially visible in the density map but cannot be modeled due to limited resolution. In addition, several serine residues (Ser12, Ser36, Ser39, Ser42) in the NTD were identified as phosphorylation sites[67]. Only Ser12 were visible in the density map. The other serine residues are located in a disordered loop that cannot be visualized.

Interactions of BTX-B with lipid

Both BTX-B sites IIB_1 and IIB_2 were found in the fenestration tunnels that are often pre-occupied by unknown lipids or molecules of hydrophobic nature. Despite the high binding affinity for BTX-B and our attempts to classify the EM particles to a homogeneous class, these particles probably still contained some residual lipids, giving rise to overlap mixture of the density where weak elongated density extended beyond the boundary of BTX-B. It is also likely that BTX-B carries lipid with it or interact with the membrane due to its hydrophobic nature. Both BTX-B from sites IIB_1 and IIB_2 rest in the fenestration with the BTX-A core facing outward toward the membrane (Fig. 1f). The BTX-A core of the BTX-B at site IIB_2 appears to interact with the acyl chain of a phospholipid from the outer leaflet membrane (Supplementary Fig. 4a) while the BTX-A core from site IIB_1 interacts with the aliphatic tail of a nearby cholesterol hemi-succinate (CHS) also from the outer leaflet membrane (Supplementary Fig. 4b). There is also a long tube

of density likely from an acyl chain of a phospholipid that connects BTX-B site IIB_1 to the inner leaflet membrane (Supplementary Fig. 4b). It is unclear whether the interactions by BTX-B with lipids have any functional role in channel activation.

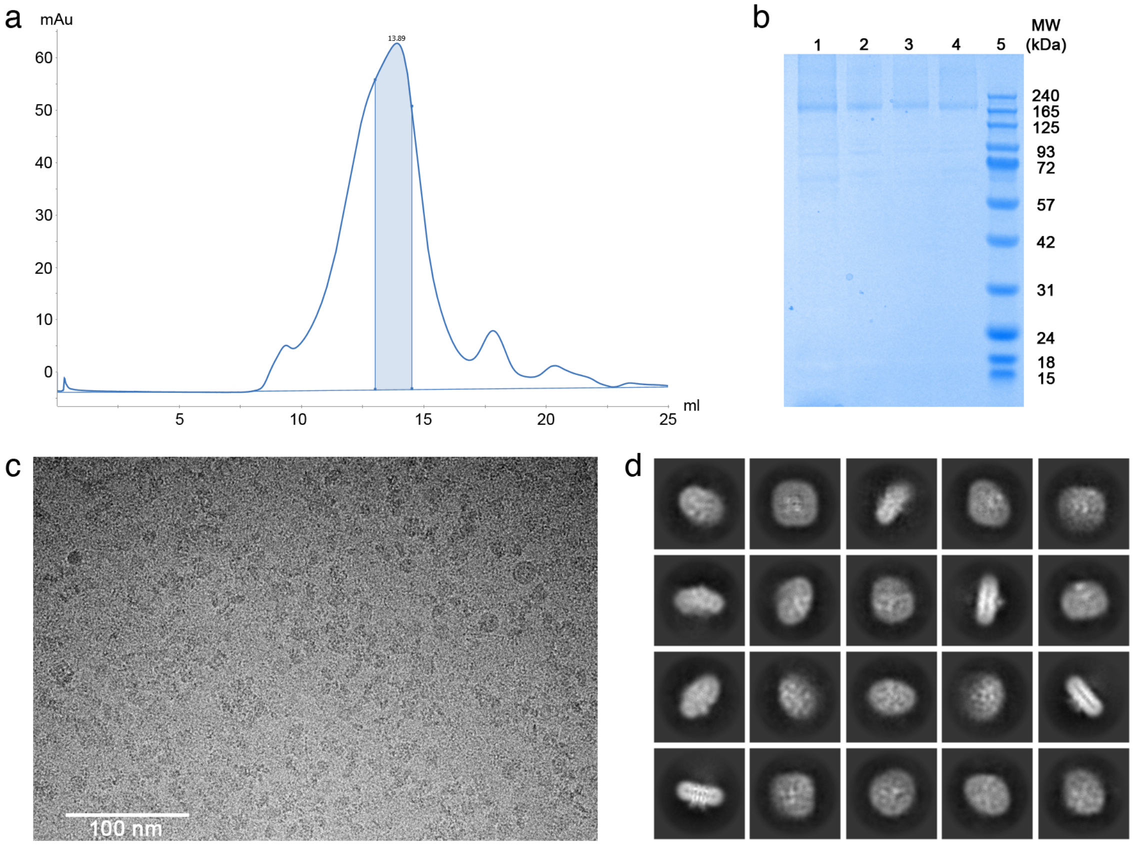

Supplementary Fig. 1. Biochemical sample preparation and electron microscopy of BTX-B bound rNa$_V$1.5c. a. Final size-exclusion chromatography (SEC2) of rNa$_V$1.5c co-purified with 600 nM BTX-B. Peak fractions used for electron microscopy are highlighted in blue. **b.** SDS-PAGE of samples from different steps of protein purification. Lane 1: FLAG elution, Lane 2: peak fractions from SEC1 with added BTX-B and LqhIII, Lane 3, peak fractions from SEC2 as in panel **a**; Lane 4: concentrated sample used for cryo-EM, Lane 5: protein ladder with molecular weights indicated on the side. **c.** A representative electron micrograph of BTX-B bound to rNa$_V$1.5c. 7,542 micrographs in total were collected. **d**. 2D classification of cryo-EM single particles.

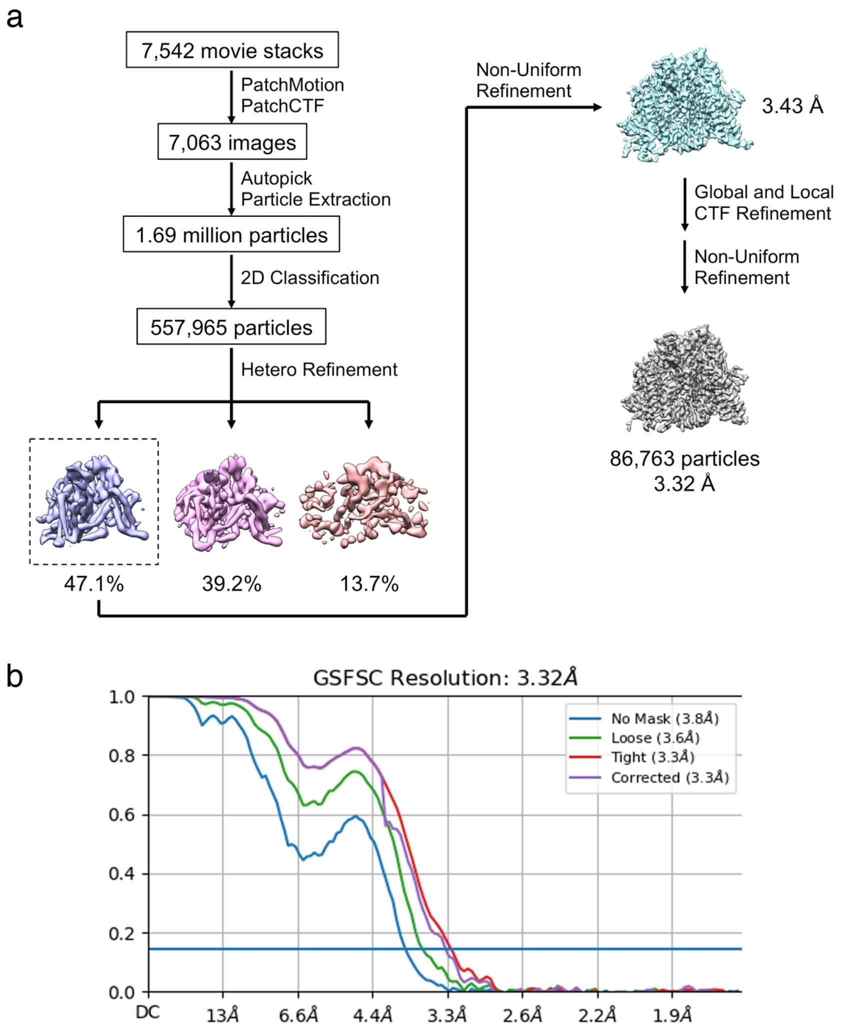

Supplementary Fig. 2. Cryo-EM data processing and analysis of BTX-B bound rNa$_V$1.5c. a. Data processing flowchart for data processing with CryoSPARC™. **b**. Gold standard Fourier Shell Correlation (FSC) plots. The final map has an overall resolution of 3.3Å (purple) using to the FSC criterion of 0.143 (blue horizontal line).

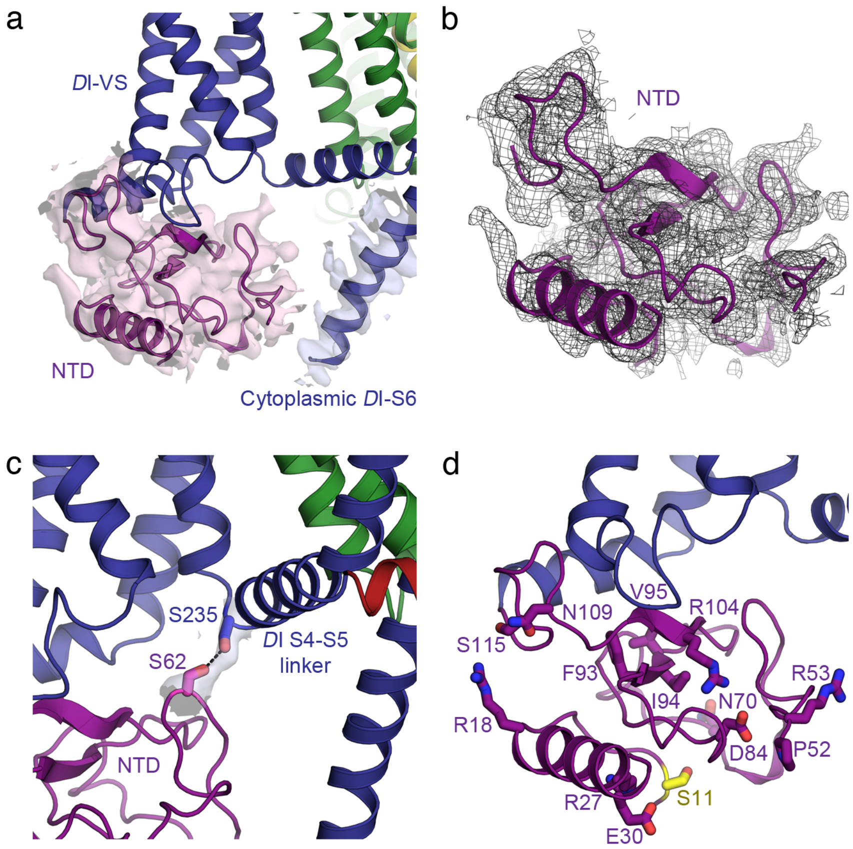

Supplementary Fig. 3. The N-terminal domain (NTD) in the structure of BTX-B bound rNa_V1.5c. a. The cytosolic S6 helix of *D*I (dark blue) appears to interact with and stabilize the NTD (dark purple). Cryo-EM density map (4σ) of the NTD (pink) and the cytosolic *D*I-S6 (light blue) are overlaid. **b.** Close-up view of the Cryo-EM density map (4σ) for the NTD (black mesh) overlaid with the NTD (dark purple). **c**. The NTD S62 (magenta sticks) forms a hydrogen bond (black dash) with S235 (blue sticks) in the *D*I S4-S5 linker to stabilize the activated conformation. Cryo-EM density (gray, 4σ) is overlaid. **d**. Close-up view of the NTD showing residues with known mutations that cause cardiac arrhythmia (magenta sticks) and known phosphorylation sites (yellow sticks) in human Na_V1.5.

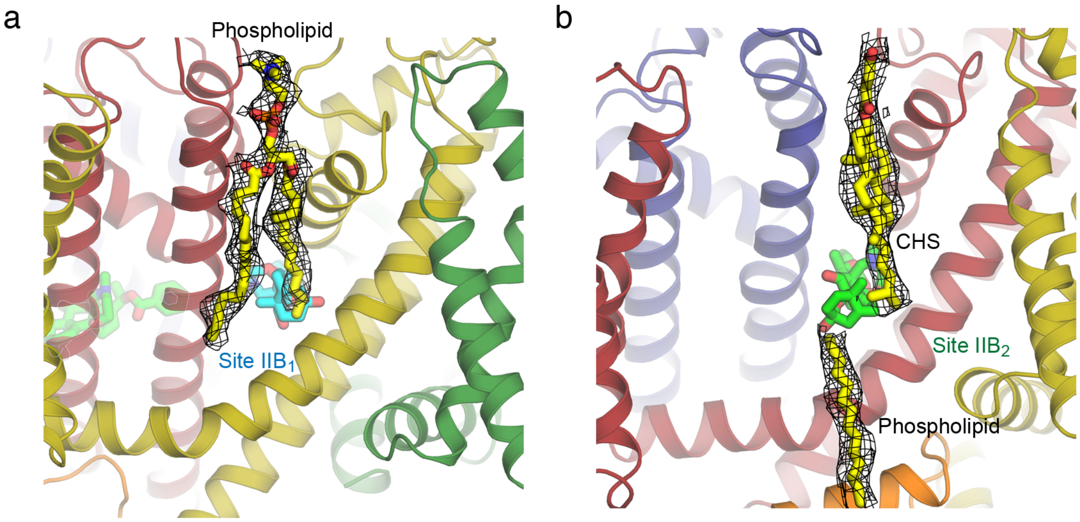

Supplementary Fig. 4. BTX-B interactions with lipids. a. Site IIB$_2$ (cyan sticks) interactions with phospholipid (bright yellow sticks). The phospholipid is located in the outer leaflet of the membrane in the outer pore region between *D*III (dark yellow) and *D*IV (dark red). Cryo-EM density (4σ) of the lipid is shown as black mesh. **b.** Site IIB$_1$ (bright green sticks) interactions with cholesterol hemi-succinate and probable phospholipid (bright yellow sticks). The cholesterol hemi-succinate is located in the outer leaflet of the membrane in the outer pore region between *D*I (dark blue) and *D*IV (dark red) while the probable phospholipid is in the inner leaflet of the membrane near the S4-S5 linker of *D*IV and the *D*III-*D*IV linker (orange). Cryo-EM density (4σ) of the lipids is shown as black mesh.

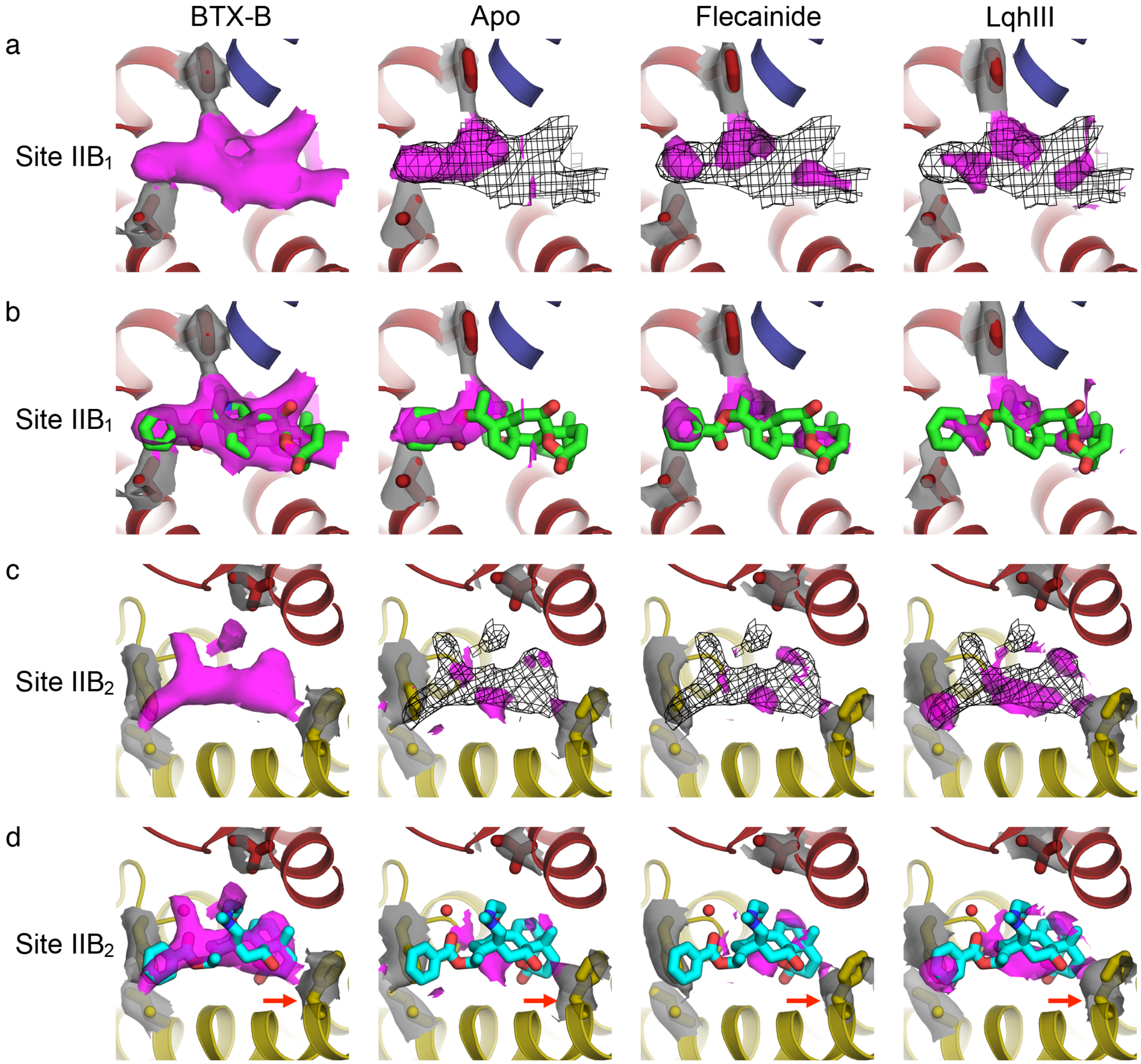

Supplementary Fig. 5. Comparison of cryo-EM density maps in the putative BTX-B binding sites from related rNa$_V$1.5c structures. The putative density for BTX-B (5σ) and density found in the same area in other cryo-EM maps are highlighted in magenta and contoured at similar levels. The high contour level of the density maps was chosen to distinguish signals from noise. Density for nearby side chains is shown in gray as control to ensure that the maps are compared at similar levels. The coordinates of rNa$_V$1.5c/BTX-B structure are overlaid with the density maps in all panels. From left to right: cryo-EM density of the BTX-B bound (this study, 5σ), Apo (EMD-20951, 4σ), Flecainide-bound (EMD-20949, 5σ), LqhIII-bound (EMD-22621, 5σ) structures. **a.** Site IIB$_1$. The putative density for BTX-B is shown as black mesh for the comparison with other density maps. **b.** Site IIB$_1$ with BTX-B (bright green sticks) overlaid. **c.** Site IIB$_2$. The putative density for BTX-B is shown as black mesh for the comparison with other density

maps. **d**. Site IIB_2 with BTX-B (cyan sticks) overlaid. Red arrows indicate density for F1467 (gray) in different rotamers. The binding of BTX-B causes the F1467 side chain to rotate away (left panel). In other remaining panels, F1467 would clash with BTX-B.

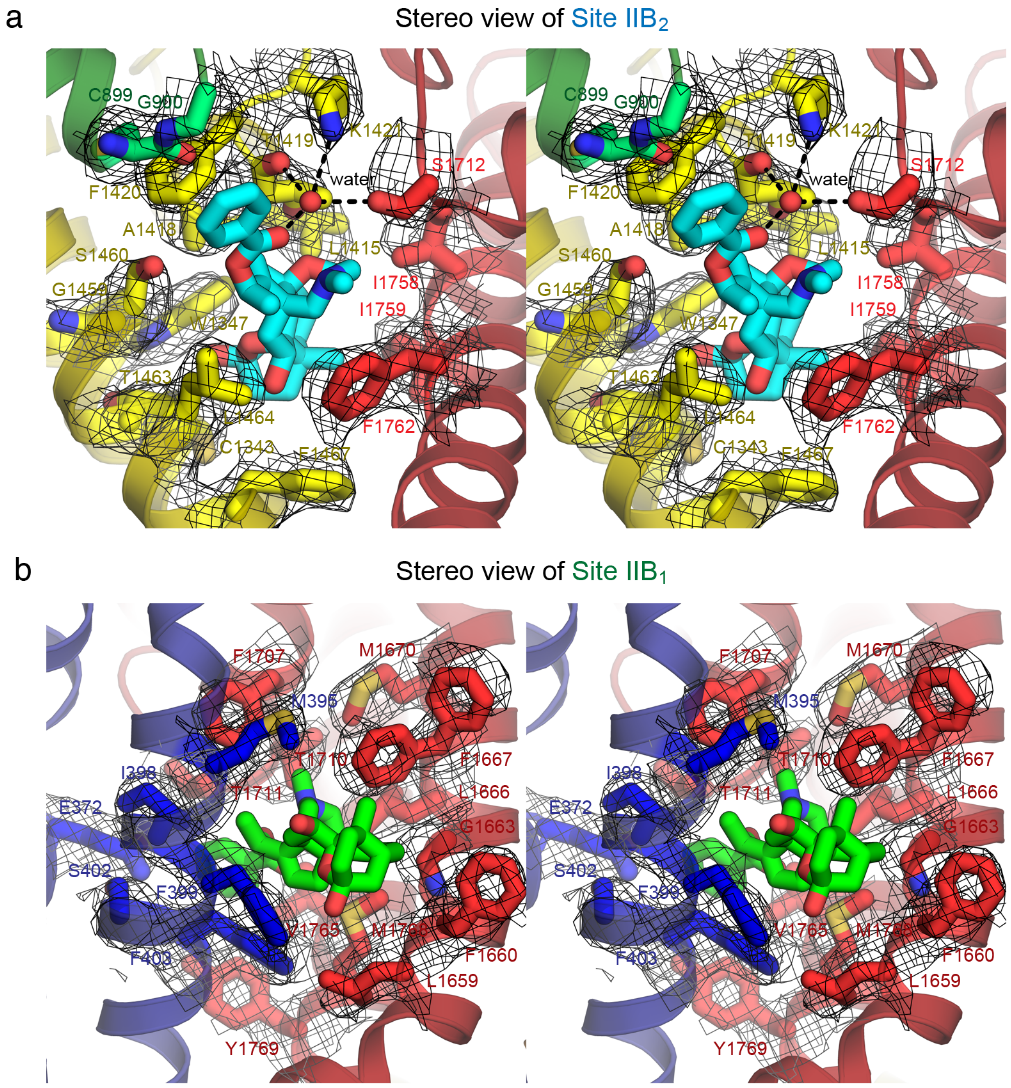

Supplementary Fig. 6. Close-up views of the Site IIB$_1$ and Site IIB$_2$. a. Stereo view of Site IIB$_2$ (cyan sticks). Amino acid residues that form the binding site are shown as sticks colored according to their domains (*D*II – lime green, *D*III – yellow, *D*IV – red) overlaid with the cryo-EM density map (black mesh, 5σ). **b.** Stereo view of Site IIB$_1$ (bright green sticks). Amino acid residues that form the binding site are shown as sticks colored according to their domains (*D*I – blue, *D*IV – red) overlaid with the cryo-EM density map (black mesh, 5σ).

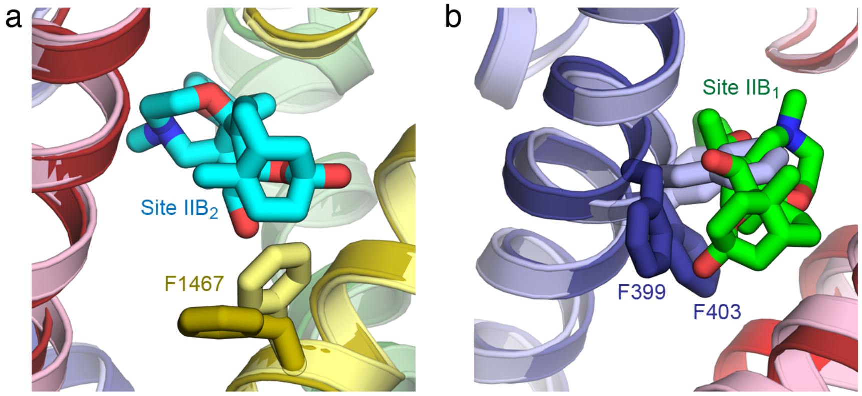

Supplementary Fig. 7. Conformational differences in the side chain rotamers between the BTX-B bound rNa$_V$1.5c and the rNa$_V$1.5c QQQ in the open state. a. Close-up view of the conformational change in the side chain rotamer of F1467 in *D*III-S6 ("down" in BTX-B bound – dark yellow vs. "up" in the open state – light yellow). Site IIB$_2$ is shown as cyan sticks. **b**. Close-up view of the conformational changes in the side chain rotamer of F399 and F403 in *D*I-S6. The side chain of F399 and F403 are "down" in the BTX-B bound structure (dark blue) but are "up" in the open state structure (light blue). Site IIB$_1$ is shown as bright green sticks.

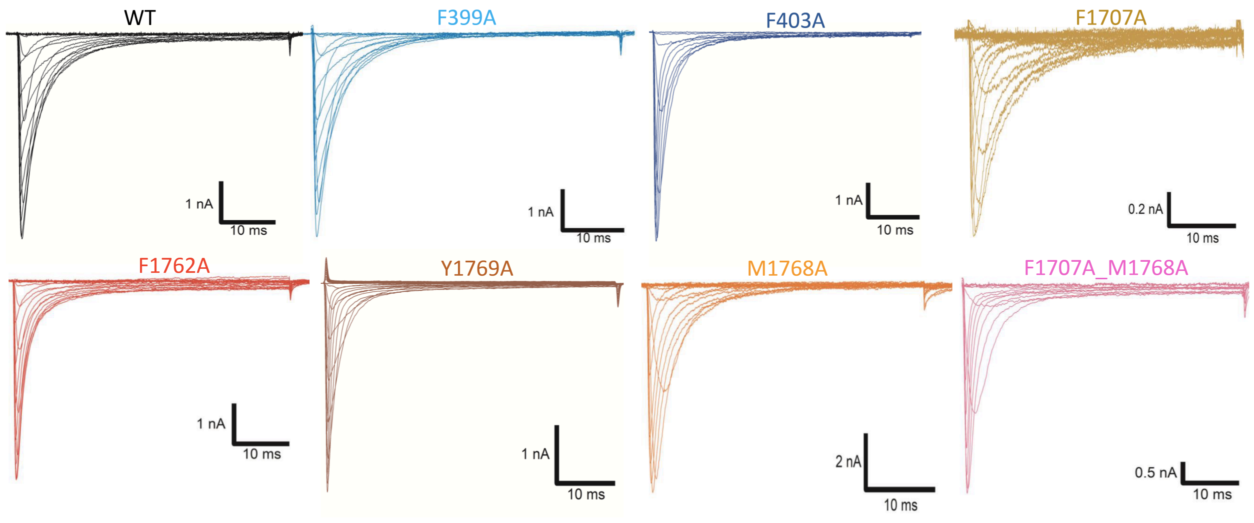

Supplementary Fig. 8. Representative current traces of rNa_V1.5c WT and mutants used in this study. Cells were held at −140 mV, and 50-ms depolarizing pulses were applied in 10-mV steps from −100 mV to 0 mV.

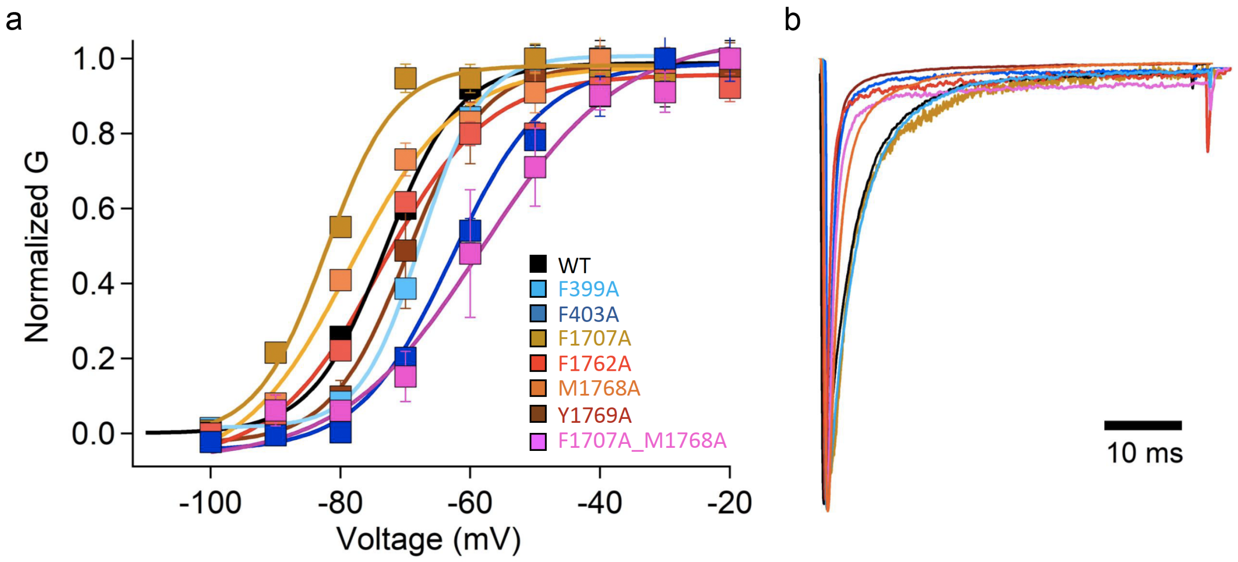

Supplementary Fig. 9. Voltage-dependence activation of rNa$_V$1.5c WT and mutants. a. Normalized conductance/voltage (G/V) curves were calculated from the I/V curves and fit with a single Boltzmann equation: $1/(1+\exp(V_{1/2}-V_m)/k)$ in which V_m is the stimulus potential, $V_{1/2}$ is the half-activation voltage, and k is a slope factor: WT, $V_{1/2}$ = $V_{1/2}$ = −67 ± 2 mV, k = 5.6 ± 0.6 mV (black squares; n = 7); F399A, $V_{1/2}$ = −67.6 ± 0.2 mV, k = 4.6 ± 0.16 mV (light blue; n = 7); F403A, $V_{1/2}$ = −67.6 ± 0.2 mV, k = 4.6 ± 0.16 mV (navy blue, n = 5); F1707A, $V_{1/2}$ = −82 ± 4 mV, k = 5.3 ± 2.9 mV (light brown; n = 7); F1762A, $V_{1/2}$ = −73.7 ± 0.9 mV, k = 8.2 ± 3.9 mV (red; n = 8), M1768A, $V_{1/2}$ = −78.5 ± 1.9 mV, k = 8.2 ± 0.9 mV (orange; n = 8), Y1769A, $V_{1/2}$ = −69 ± 0.6 mV, k = 5.8 ± 3.3 mV (red; n = 8), F1707A_ M1768A, $V_{1/2}$ = −58.2 ± 0.9 mV, k = 10.75 ± 2.5 mV (pink; n = 8) The activation curves were constructed from I–V curves. Data are presented as mean values ± SEM. **b.** Overlay of representative peak current traces showing persistent current of mutants compared with rNa$_V$1.5c WT (same color codes as in panel **a**).

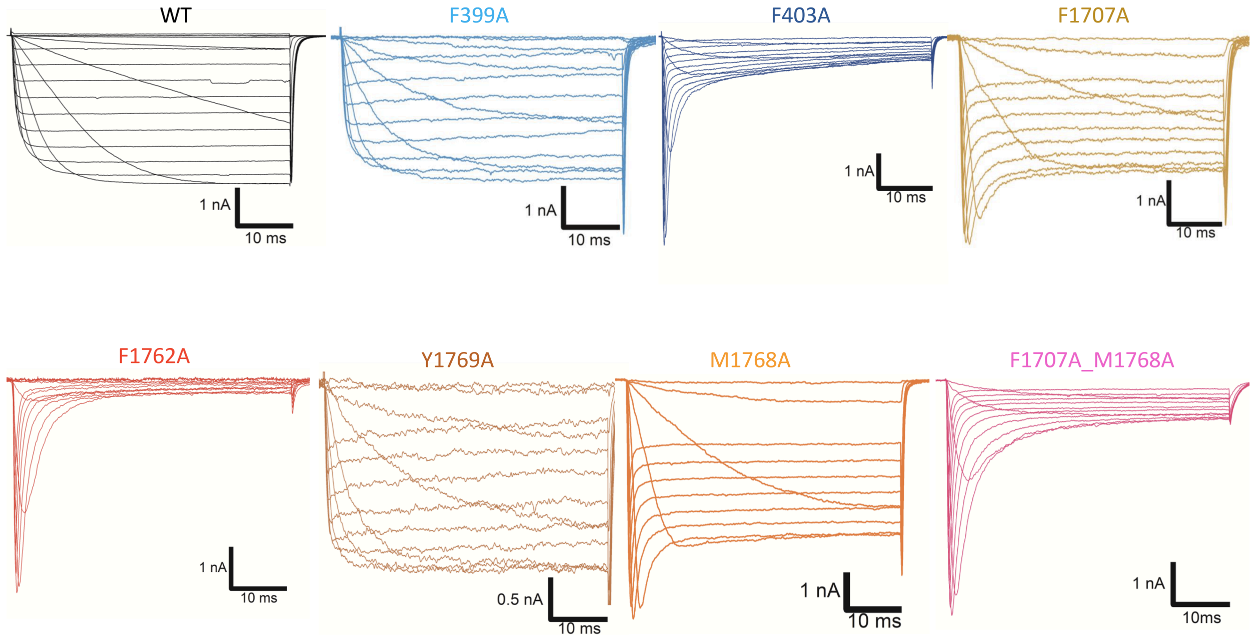

Supplementary Fig. 10. Representative current traces of rNa$_V$1.5c WT and mutants after exposure to 10 μM BTX-B. Cells were held at −140 mV, and 50-ms depolarizing pulses were applied in 10-mV steps from −100 mV to 0 mV.

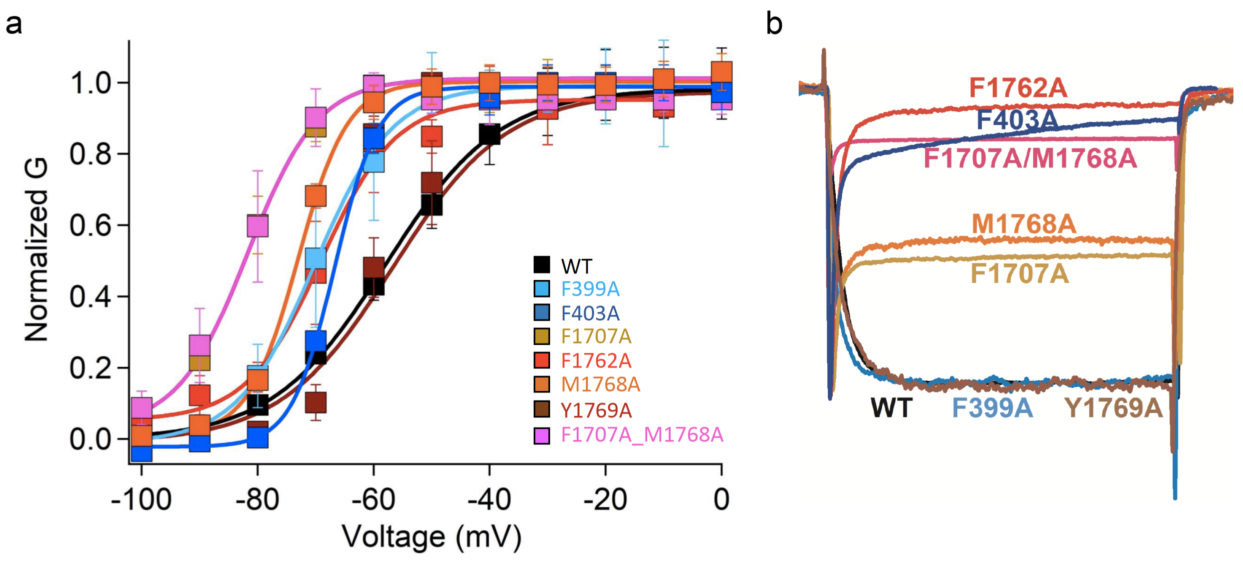

Supplementary Fig. 11. Voltage dependence activation of rNa$_V$1.5c WT and mutants in the presence of 10 μM BTX-B. a. Normalized conductance/voltage (G/V) curves were calculated from the I/V curves and fit with a single Boltzmann equation: $1/(1+\exp(V_{1/2}-V_m)/k)$ in which V_m is the stimulus potential, $V_{1/2}$ is the half-activation voltage, and k is a slope factor: WT, $V_{1/2}$ = −69 ± 0.9 mV, k = 6.2 ± 2.8 mV (black squares; n = 7); F399A, $V_{1/2}$ = −70 ± 2 mV, k = 6.6 ± 2.8 mV (light blue; n = 7); F403A, $V_{1/2}$ = −66.7 ± 2 mV, k = 3.7 ± 3 mV (navy blue; n = 5); F1707A, $V_{1/2}$ = −82 ± 4 mV, k = 6.1 ± 2.9 mV (light brown; n = 7); F1762A, $V_{1/2}$ = −69 ± 0.9 mV, k = 6.4 ± 3.9 mV (red; n = 8), M1768A, $V_{1/2}$ = −73 ± 0.9 mV, k = 4.3 ± 0.9 mV (orange; n = 8), Y1769A, $V_{1/2}$ = −57 ± 0.9 mV, k = 9.7 ± 3.3 mV (red; n = 8), F1707A_ M1768A, $V_{1/2}$ = −81.7 ± 0.9 mV, k = 6.0 ± 3.9 mV (pink; n = 8). The activation curves were constructed from I–V curves. Data are presented as mean values ± SEM. **b.** Overlay of representative peak current traces showing the effect of 10 μM BTX-B on mutants compared with rNa$_V$1.5c WT (same color codes as in panel **a**).

Supplementary Table 1. Cryo-EM data collection and model refinement statistics

**rNa$_V$1.5c/BTX-B complex**	EMDB: EMD-41071<br>PDB: 8T6L
**Data collection**	
EM equipment	Titan Krios (Thermo Fisher Scientific)
Voltage (kV)	300
Detector	Gatan K3 Summit
Energy filter	Gatan GIF Quantum, 20eV slit
Electron exposure rate (e^-/pixel/sec)	20
Electron exposure (e^-/Å^2)	60
Movies collected	7,542
Movies used	7,063
Defocus range (μm)	-0.5 to -2.0
Pixel size (Å)	0.8255 (0.41275)[a]
**3D Reconstruction**	
Software	CryoSPARC™
Symmetry	c1
Initial particle images picked	~1.69 million
Final particle images used	86,763
Map resolution (Å) at FSC = 0.143 threshold	3.3
Map resolution range (Å)	2.5 to 5.0
Map sharpening B factor (Å^2)	-86.2
**Model refinement**	
Software	PHENIX
Model vs. map correlation (Å) at FSC = 0.143/0.5	3.3/3.5
Protein residues	1,237
R.m.s. deviations	
Bond length (Å)	0.002
Bond angel (°)	0.556
Model validation	
MolProbity score	1.67
Clashscore	6.61
Rotamers ourliers (%)	0.00
Ramachandran plot	
Favored (%)	95.60
Allowed (%)	4.40
Outliers (%)	0.00

[a]Super-resolution pixel size

Supplementary Table 2. Summary of residues forming the BTX-B binding sites in rNa$_V$1.5 and their clinical variants in human Na$_V$1.5

Location	Residue in rNa$_V$1.5	Residue in human Na$_V$1.5	Clinical variant	Disease
**Site IIB$_1$**				
*D*I-S5	Q372	Q371		
*D*I-S6	M395	M394		
	I398	I397	I397T*	LQT3
	F399	F398		
	S402	S401		
	F403	F402		
*D*IV-S5	L1659	L1657		
	F1660	F1658		
	G1663	G1661	G1661R*	BRGDA1
	L1666	L1664		
	F1667	F1665		
*D*IV P-loop	F1707	F1705	F1705S	SIDS
	T1710	T1708		
	T1711	T1709	T1709M/R*	BRGDA1
*D*IV-S6	V1765	V1763	V1763M	LQT3
	M1768	M1766	M1766L	LQT3
	Y1769	Y1767	Y1767C*	LQT3
**Site IIB$_2$**				
*D*II P-loop	C899	C896	C896S	BRGDA1
	G900	G897		
*D*III-S5	C1343	C1341		
	W1347	W1345		
*D*III P-loop	L1415	L1413		
	A1418	A1416		
	T1419	T1417		
	F1420	F1418		
	K1421	K1419	K1419E*	BRGDA1
*D*III-S6	G1459	G1457		
	S1460	S1458	S1458Y	LQT3
	T1463	T1461		
	L1464	L1462		
	F1467	F1465		
*D*IV P-loop	S1712	S1710	S1710L	VF1
*D*IV-S6	I1758	I1756		
	I1759	I1757		
	F1762	F1760		

Asterisk (*) denotes unknown pathological significance.

Abbreviations:

LQT3 = Long QT syndrome 3

BRGDA1 = Brugada syndrome 1

SIDS = Sudden infant death syndrome

VF1 = Familial paroxysmal ventricular fibrillation 1

Supplementary Table 3. Summary of inhibition ratios R_i for rNa$_V$1.5c WT and mutants in the presence of 10 μM BTX-B

**rNa$_V$1.5c**	**R_i[a]**
WT	0.78 ± 0.04
F399A	0.78 ± 0.06
F403A	0.23 ± 0.03
F1707A	0.61 ± 0.35
F1762A	0.20 ± 0.03
M1768A	0.55 ± 0.04
Y1769A	0.77 ± 0.07
F1707A_M1768A	0.32 ± 0.05

[a]R_i measures the sodium current magnitude at 40 ms after the start of the depolarizing pulse (I_{40ms}) divided by the amplitude of the peak current (I_{peak}) from the same trace. Data are presented as mean values ± SEM.

Supplementary Table 4. Summary of mutations in the dual BTX receptor sites in other voltage-gated sodium channels

Location	Mutations	Na_V channel	Residue in $rNa_V1.5c$	Effect of BTX	References
**Site IIB_1**					
*D*I-S6	S401K/R	$hNa_V1.5$	S402	BTX resistant	Wang et al., 2006
	F430K	$rNa_V1.4$	F403	BTX sensitive	Wang & Wang, 1998
	I433K	$rNa_V1.4$	V406	BTX resistant	Wang & Wang, 1998
	N434K	$rNa_V1.4$	N407	BTX resistant	Logan et al., 2006
	L437K	$rNa_V1.4$	L410	BTX resistant	Wang & Wang, 1998
*D*IV-S6	V1583C	$rNa_V1.4$	V1766	Blockage of cysteine modification	Vedantham & Cannon, 2000
	N1584A/D/K	$rNa_V1.4$	N1767	BTX resistant	Wang & Wang, 1999; Logan et al., 2006
	Y1771A	$rNa_V1.2$	Y1769	BTX sensitive	Linford et al., 1998
	Y1586A/K	$rNa_V1.4$	Y1769	BTX sensitive	Wang & Wang, 1999
**Site IIB_2**					
*D*III P-loop	F1236K/R	$rNa_V1.4$	F1420	BTX resistant	Wang et al., 2006; Logan et al., 2006
*D*III-S6	S1276K	$rNa_V1.4$	S1460	BTX resistant	Wang et al., 2000
	L1280K	$rNa_V1.4$	L1464	BTX resistant	Wang et al., 2000; Logan et al., 2006
*D*IV-S6	I1760A	$rNa_V1.2$	I1758	Reduced BTX sensitivity	Linford et al., 1998
	F1764A	$rNa_V1.2$	F1762	BTX resistant	Linford et al., 1998
	F1710A/I	$rNa_V1.3$	F1762	BTX resistant	Li et al., 2002
	F1579K	$rNa_V1.4$	F1762	BTX resistant	Wang & Wang, 1999; Logan et al., 2006
