# Supplementary material for: Dual receptor-sites reveal the structural basis for hyperactivation of sodium channels by poison-dart toxin batrachotoxin
Source: Nat Commun. 2024 Mar 14;15:2306. doi: 10.1038/s41467-024-45958-w (PMC10940626; doi:10.1038/s41467-024-45958-w)
Supplement: Supplementary file 3 — Description of Additional Supplementary Files [file 41467_2024_45958_MOESM3_ESM.pdf]

Description of Additional Supplementary Materials

File Name: Supplementary Movie 1.

Description: Close-up view of the receptor sites for BTX-B site IIB2. BTX-B site DIII/DIV is shown as cyan stick. Amino acid residues that form the binding site are shown as sticks colored according to their domains (DII – lime green, DIII- yellow, DIV – red) overlaid with the cryo-EM density map (black mesh, 5σ).

File Name: Supplementary Movie 2.

Description: Close-up view of the receptor sites for BTX-B sites IIB1. BTX-B site DI/DIV is shown as bright green stick. Amino acid residues that form the binding site are shown as sticks colored according to their domains (DI – blue, DIV – red) overlaid with the cryo-EM density map (black mesh, 5σ).

File Name: Supplementary Data 1.

Description: List of mutagenesis primers.
