# Supplementary material for: Dual receptor-sites reveal the structural basis for hyperactivation of sodium channels by poison-dart toxin batrachotoxin
Source: Nat Commun. 2024 Mar 14;15:2306. doi: 10.1038/s41467-024-45958-w (PMC10940626; doi:10.1038/s41467-024-45958-w)
Supplement: Supplementary file 7 — Reporting Summary [file 41467_2024_45958_MOESM7_ESM.pdf]

nature portfolio

Corresponding author(s): William A. Catterall

Last updated by author(s): Jan 25, 2024

Reporting Summary

Nature Portfolio wishes to improve the reproducibility of the work that we publish. This form provides structure for consistency and transparency in reporting. For further information on Nature Portfolio policies, see our Editorial Policies and the Editorial Policy Checklist.

Statistics

For all statistical analyses, confirm that the following items are present in the figure legend, table legend, main text, or Methods section.

n/a	Confirmed	
☐	☒	The exact sample size (*n*) for each experimental group/condition, given as a discrete number and unit of measurement
☒	☐	A statement on whether measurements were taken from distinct samples or whether the same sample was measured repeatedly
☐	☒	The statistical test(s) used AND whether they are one- or two-sided<br>*Only common tests should be described solely by name; describe more complex techniques in the Methods section.*
☒	☐	A description of all covariates tested
☒	☐	A description of any assumptions or corrections, such as tests of normality and adjustment for multiple comparisons
☐	☒	A full description of the statistical parameters including central tendency (e.g. means) or other basic estimates (e.g. regression coefficient) AND variation (e.g. standard deviation) or associated estimates of uncertainty (e.g. confidence intervals)
☐	☒	For null hypothesis testing, the test statistic (e.g. *F*, *t*, *r*) with confidence intervals, effect sizes, degrees of freedom and *P* value noted<br>*Give P values as exact values whenever suitable.*
☒	☐	For Bayesian analysis, information on the choice of priors and Markov chain Monte Carlo settings
☒	☐	For hierarchical and complex designs, identification of the appropriate level for tests and full reporting of outcomes
☒	☐	Estimates of effect sizes (e.g. Cohen's *d*, Pearson's *r*), indicating how they were calculated

Our web collection on statistics for biologists contains articles on many of the points above.

Software and code

Policy information about availability of computer code

Data collection	Pulse version 8.5 for electrophysiology; SerialEM version 4.0 for cryo-EM
Data analysis	Igor Pro version 6.37 for electrophysiology; CryoSPARC version 3.3 for cryo-EM

For manuscripts utilizing custom algorithms or software that are central to the research but not yet described in published literature, software must be made available to editors and reviewers. We strongly encourage code deposition in a community repository (e.g. GitHub). See the Nature Portfolio guidelines for submitting code & software for further information.

Data

Policy information about availability of data

All manuscripts must include a data availability statement. This statement should provide the following information, where applicable:

- Accession codes, unique identifiers, or web links for publicly available datasets
- A description of any restrictions on data availability
- For clinical datasets or third party data, please ensure that the statement adheres to our policy

The cryo-EM map has been deposited in the Electron Microscopy Data Bank (EMDB) under accession code EMD-41071 [https://www.ebi.ac.uk/pdbe/entry/emdb/EMD-41071] (the rNav1.5c/BTX-B complex). The atomic coordinates have been deposited in the Protein Data Bank (PDB) under accession code 8T6L [https://doi.org/10.2210/pdb8T6L/pdb] (the rNav1.5c/BTX-B complex).
Additional structures used in this study:

PDB 3UZ0 [http://doi.org/10.2210/pdb3UZ0/pdb]
PDB 3UZ3 [http://doi.org/10.2210/pdb3UZ3/pdb]
PDB 6LQA [http://doi.org/10.2210/pdb6LQA/pdb]
PDB 7FBS [http://doi.org/10.2210/pdb7FBS/pdb]
PDB 7K18 [http://doi.org/10.2210/pdb7K18/pdb]
PDB 7W77 [http://doi.org/10.2210/pdb7W77/pdb]
PDB 7XM9 [http://doi.org/10.2210/pdb7XM9/pdb]
The source data underlying Figures 1d and 4d, and Supplementary Fig. 1 are provided as a Source Data file.

Research involving human participants, their data, or biological material

Policy information about studies with human participants or human data. See also policy information about sex, gender (identity/presentation), and sexual orientation and race, ethnicity and racism.

Reporting on sex and gender	N/A
Reporting on race, ethnicity, or other socially relevant groupings	N/A
Population characteristics	N/A
Recruitment	N/A
Ethics oversight	N/A

Note that full information on the approval of the study protocol must also be provided in the manuscript.

Field-specific reporting

Please select the one below that is the best fit for your research. If you are not sure, read the appropriate sections before making your selection.

☒ Life sciences ☐ Behavioural & social sciences ☐ Ecological, evolutionary & environmental sciences

For a reference copy of the document with all sections, see nature.com/documents/nr-reporting-summary-flat.pdf

Life sciences study design

All studies must disclose on these points even when the disclosure is negative.

Sample size	Cells were chosen between 3 and 8 for electrophysiology experiments based on previous results with similar methods and determination of the expected standard error (Jiang et al., 2021, Cell 184, 5151–5162). The number of cryo-EM micrographs collected were chosen based on previous results with similar methods and determination of the expected resolution (Jiang et al., 2021, Cell 184, 5151–5162).
Data exclusions	No electrophysiology data were excluded. Cryo-EM micrographs with CTF estimation worse than 6 Angstrom were excluded.
Replication	Electrophysiology experiments were replicated 3-8 times.
Randomization	Cells for electrophysiology studies were allocated into the same group (wild-type vs. different mutants) based on the predefined expression by baculovirus infection. From there, cells in the same group were selected randomly from experimental cultures of many cells and investigated by whole-cell patch clam electrophysiology. For Cryo-EM, particles were not picked randomly. The Blob picker algorithm picked particles based on predefined diameters.
Blinding	Blinding was not possible for structural biology because the identity of the purified protein is known. In addition, cryo-EM particles and class averages are selected based on previously known features. Blinding was not possible for electrophysiology because different cell cultures expressed predefined wild-type or mutants.

Reporting for specific materials, systems and methods

We require information from authors about some types of materials, experimental systems and methods used in many studies. Here, indicate whether each material, system or method listed is relevant to your study. If you are not sure if a list item applies to your research, read the appropriate section before selecting a response.

Materials & experimental systems

n/a	Involved in the study
☒	☐ Antibodies
☐	☒ Eukaryotic cell lines
☒	☐ Palaeontology and archaeology
☒	☐ Animals and other organisms
☒	☐ Clinical data
☒	☐ Dual use research of concern
☒	☐ Plants

Methods

n/a	Involved in the study
☒	☐ ChIP-seq
☒	☐ Flow cytometry
☒	☐ MRI-based neuroimaging

Eukaryotic cell lines

Policy information about cell lines and Sex and Gender in Research

Cell line source(s)	Sf9 (Spodoptera frugiperda) insect cells (ThermoFisher Scientific, catalogue no. B82501)<br>Human HEK293S GnTI– cells (female, American Type Culture Collection, catalogue no. CRL-3022)
Authentication	The cell lines were not authenticated.
Mycoplasma contamination	The cell lines were not tested for Mycoplasma contamination.
Commonly misidentified lines<br>(See ICLAC register)	No commonly misidentified lines were used in the study.

Plants

Seed stocks	*Report on the source of all seed stocks or other plant material used. If applicable, state the seed stock centre and catalogue number. If plant specimens were collected from the field, describe the collection location, date and sampling procedures.*
Novel plant genotypes	*Describe the methods by which all novel plant genotypes were produced. This includes those generated by transgenic approaches, gene editing, chemical/radiation-based mutagenesis and hybridization. For transgenic lines, describe the transformation method, the number of independent lines analyzed and the generation upon which experiments were performed. For gene-edited lines, describe the editor used, the endogenous sequence targeted for editing, the targeting guide RNA sequence (if applicable) and how the editor was applied.*
Authentication	*Describe any authentication procedures for each seed stock used or novel genotype generated. Describe any experiments used to assess the effect of a mutation and, where applicable, how potential secondary effects (e.g. second site T-DNA insertions, mosiacism, off-target gene editing) were examined.*
